# Automatically Constructed Neural Network Potentials for Molecular Dynamics Simulation of Zinc Proteins

**DOI:** 10.3389/fchem.2021.692200

**Published:** 2021-06-18

**Authors:** Mingyuan Xu, Tong Zhu, John Z. H. Zhang

**Affiliations:** ^1^ Shanghai Engineering Research Center of Molecular Therapeutics and New Drug Development, Shanghai Key Laboratory of Green Chemistry and Chemical Process, School of Chemistry and Molecular Engineering, East China Normal University, Shanghai, China; ^2^ NYU-ECNU Center for Computational Chemistry at NYU Shanghai, Shanghai, China; ^3^ Department of Chemistry, New York University, New York, NY, United States; ^4^ Collaborative Innovation Center of Extreme Optics, Shanxi University, Taiyuan, China

**Keywords:** force field, neural network, zinc protein, molecular dynamic simulation, metalloproteins

## Abstract

The development of accurate and efficient potential energy functions for the molecular dynamics simulation of metalloproteins has long been a great challenge for the theoretical chemistry community. An artificial neural network provides the possibility to develop potential energy functions with both the efficiency of the classical force fields and the accuracy of the quantum chemical methods. In this work, neural network potentials were automatically constructed by using the ESOINN-DP method for typical zinc proteins. For the four most common zinc coordination modes in proteins, the potential energy, atomic forces, and atomic charges predicted by neural network models show great agreement with quantum mechanics calculations and the neural network potential can maintain the coordination geometry correctly. In addition, MD simulation and energy optimization with the neural network potential can be readily used for structural refinement. The neural network potential is not limited by the function form and complex parameterization process, and important quantum effects such as polarization and charge transfer can be accurately considered. The algorithm proposed in this work can also be directly applied to proteins containing other metal ions.

## Introduction

Zinc ions are important protein cofactors and play important roles in maintaining the structural stability of proteins, signal transduction, and enzyme catalysis. There is plenty of evidence that shows that zinc-containing proteins are associated with many human diseases, such as cancer, rheumatism, and Alzheimer’s disease. Since the d-orbital of Zn^2+^ is fulfilled with electrons, its coordination mode is very flexible. In aqueous solutions, Zn^2+^ and water molecules can form an octahedral six-coordinated complex. In proteins, Zn^2+^ usually forms a tetrahedral four-coordinate complex with cysteine, histidine, and aspartic/glutamic acids. Molecular dynamics simulation based on empirical potential energy functions (force fields) is one of the main theoretical methods to study the structure and dynamic properties of zinc-containing proteins. Unfortunately, most existing force fields are generally incapable of properly describing the interactions between metal ions and proteins. In most cases, one uses a charged ball to represent zinc ions. Its interaction with other molecules is described by electrostatic and van der Waals potentials. However, a series of works have found that this treatment is problematic. In a recent study of Ahlstrand et al. (Ahlstrand et al., 2017), 2ns MD simulation for a zinc-protein S100A12 was performed with the CHARMM27 force field. After simulation, the coordination mode of Zn^2+^ changed from a tetrahedral structure composed of three imidazole rings and a carboxyl group in the crystal structure to a six-coordinate structure with two water molecules squeezed into the metal binding group. The same phenomenon was also found when we simulated the matrix metalloproteinase three using the Amber ff99SB force field. It looked like these two force fields overestimated the interaction between Zn^2+^ and negatively charged groups in protein. Ahlstrand et al. also evaluated the interaction energies between Zn and its ligands in complexes that mimic protein binding sites using quantum mechanics (QM) and several force fields. The calculated results show that non-polarizable force fields cannot reproduce even the relative order of the QM interaction energies. Nowadays, it has been widely accepted that it is impossible to use only electrostatic and van der Waals terms to correctly simulate the interaction between Zn^2+^ and proteins. Quantum effects, especially polarization and charge transfer must be considered. In the past two decades, several polarizable force fields for zinc proteins were developed, such as the SIBFA model of Gresh et al. (Gresh, 1995; Gresh et al., 2011), the CTPOL model of Lim et al. (Sakharov and Lim, 2005; Sakharov and Lim, 2009), the SLEF model of Wu et al. (Li and Merz, 2014), the AMOEBA model of Ren et al. (Wu et al., 2010; Wu et al., 2011), the 12–6–4 LJ-type non-bonded model of Li and Merz (Li and Merz, 2014), the ABEEM of Yang et al. (Yang and Cui, 2007), the Drude oscillator model of Roux et al. (Lemkul et al., 2016), a new CT model by Rick et al. (Soniat et al., 2015), and the QPCT model developed in our previous work. (Zhu et al., 2013) Some of these force field also consider the charge transfer effect, which can be seen from their names. However, although the performance of these force fields clearly improved, (Li and Merz, 2017) this improvement is not always guaranteed.

Compared with force fields, the QM method is undoubtedly more rigorous and accurate, but its computational cost severely limits its application in large systems such as proteins. Although one can use a hybrid QM/molecular mechanics (MM) method (Cauët et al., 2010), linear-scaling and/or fragmentation QM methods (Dahlke and Truhlar, 2007a; Dahlke and Truhlar, 2007b; Dahlke and Truhlar, 2008; Liu et al., 2018) to treat larger molecular systems, the efficiency of these methods still cannot meet the needs of long-term MD simulations. Fortunately, machine learning methods, especially artificial neural networks (NNs) provide the possibility to develop molecular potentials with both the efficiency of the MM method and the accuracy of the QM method (Hansen et al., 2013). NNs constitute a very flexible and unbiased class of mathematical functions, which in principle is able to approximate any real-valued function to arbitrary accuracy. In 2007, Behler and Parrinello firstly proposed the high-dimensional neural network (HDNN) (Behler and Parrinello, 2007; Behler, 2011a; Behler, 2011b; Morawietz et al., 2012; Behler, 2017). Since then, many neural network-based force fields have been developed to simulate the dynamic properties of water, small organic molecules, and metal materials. For example, the GDML and DTNN models developed by Müller et al. (Chmiela et al., 2017; Schutt et al., 2017; Sauceda et al., 2019), the kCON model of Hammer et al. (Chen et al., 2018), and the Deep Potential method of E and co-workers (Zhang et al., 2018). Yang et al. also proposed a novel NN force field for a water system based on an electrostatically embedded two-body expansion scheme. (Wang and Yang, 2018) Currently there are several open-source packages like DeepMD-kit (Wang et al., 2018), TensorMol (Yao et al., 2017; Yao et al., 2018), and TorchMD (Doerr et al., 2021) which can train neural network potentials for specific molecular systems in a straightforward manner.

In our previous study, we also proposed a neural network potential model (NN/MM-RESP) for the hydration of zinc ion (Xu et al., 2019). This model describes the interactions between Zn^2+^ and water accurately and can reproduce the hydration structure of Zn^2+^ well in MD simulations. Recently, we proposed an ESOINN-DP (enhanced self-organizing incremental high dimensional neural network—deep potential) method that can construct a reference dataset and NN potentials for molecular systems automatically (Mingyuan et al., 2021). In this study, on the basis of these two works, we developed NN potentials specifically for zinc-containing proteins, and systematically benchmarked them, demonstrating their accuracy and efficiency. The paper is organized as follows. In *The ESOINN-DP Method* the basic algorithms of the ESOINN-DP method are briefly introduced. Then, MD simulations with NN potentials were performed for zinc proteins with four common coordination modes, and the accuracy of these models was analyzed. Finally, brief conclusions and outlooks are given in the last section.

## Theory and Method

### The NN Potential

**FIGURE 1:**
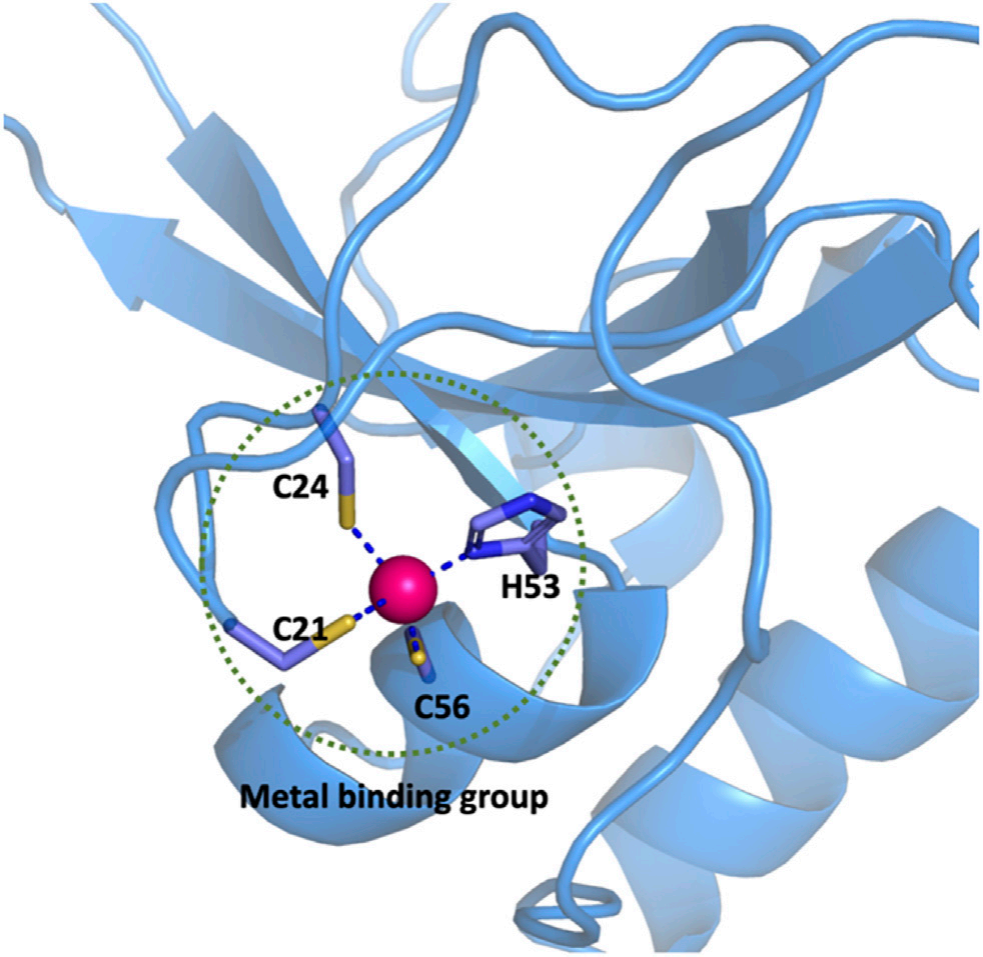
Definition of the metal binding group in a CCCH-type zinc finger protein.

In this work, if the distance between any atom of a residue that forms a coordination bond with Zn^2+^ is less than 2.8 Å, the side chain or main chain which contains the coordinated atom of this particular residue is treated as a member of the metal binding group (MBG). For example, there are three cysteine and one histidine residues that coordinate to the zinc ion in a CCCH-type zinc finger (PDB ID: 2L30) as shown in Figure 1. All the atoms shown by the ball or stick model within the dotted circle are defined as the metal binding group. According to the statistical data of MBG geometries in the PDB database, the cut-off distance of 2.8 Å was chosen because it covers metal-ligand bond distance in most common metalloproteins. Hydrogen atoms are added to saturated the MBG at the position of broken bonds.

Here, a strategy similar to the QM/MM method is used for the calculation of the total energy of a given system, which we simply named NN/MM-RESP-MBG. The potential energy and atomic forces of the entire MBG region will be predicted by the neural network, while the rest of the system is described by the classical force field. The interaction between the MBG and the other parts is described by the electrostatic and van der Waals interactions. To better describe polarization and charge transfer effects in MBG, the RESP (restrained electrostatic potential atomic partial charges) method is employed to fit the atomic charge of MBG, and then these charges are learned by the neural network model to achieve efficient prediction in the MD simulation. The total energy of the protein can then be expressed as follows:

$$\begin{array}{c} E_{total}=E_{MBG}^{NN}+E_{MM}+\sum_{i\in MBG}\sum_{j\notin MBG}\left(E_{i,j}^{ele}+E_{i,j}^{vdw}\right) \end{array}$$ (1)

### The ESOINN-DP Method

**FIGURE 2:**
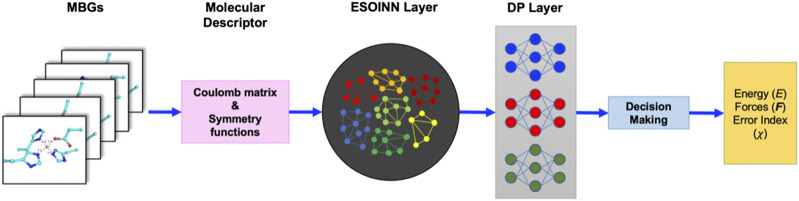
Framework of the ESOINN-DP method.

To automatically train the NN potential and NN charge model, we employed the ESOINN-DP method developed in our previous work. (Xu et al., 2021) Its framework is shown in Figure 2.

In ESOINN-DP, each MBG structure is represented by two set of molecular descriptors: the regularized sorted eigen spectrum of the Coulomb matrix (RSES) and the ANI-1 symmetry functions. The definition of the Coulomb matrix is

$$C_{ij}=\{\begin{array}{cc} 0.5Z_{i}^{2.4} & \forall i=j\\ \frac{Z_{i}Z_{j}}{|R_{i}-R_{j}} & \forall i\neq jandi\notin virtualatoms\\ 0 & \forall i\neq jandi\in virtualatoms \end{array}$$ (2)

The RSESs are used as input by ESOINN to automatically construct the reference dataset, and ESOINN can ensure that the dataset has minimal redundancy while covering the target chemical space (Furao et al., 2007; Mingyuan et al., 2021). In addition, the final dataset will be divided into several subsets according to the similarity between the MBG structure after passing through the ESOINN layer. The ANI-1 symmetry functions $S_{\alpha }$ developed by Isayev and co-workers (Smith et al., 2017) are used as descriptors to fit energy, atomic forces, and RESP charges in the DP layer. $S_{\alpha }$ consist of radial and angular parts as shown in Eqs. 3, 4.

$$\begin{array}{c} S_{\text{α}}\left(radial\right)=\sum_{j\neq i}e^{-\text{η}{\left(R_{ij}-R_{s}\right)}^{2}}f_{c}\left(R_{ij}\right) \end{array}$$ (3)

$$\begin{array}{c} S_{\text{α}}\left(angular\right)=2^{1-\text{ζ}}\sum_{j\neq i,j\neq k}{\left(1+cos\left({\text{θ}}_{ijk}-{\text{θ}}_{s}\right)\right)}^{\text{ζ}}\times e^{-\text{η}{\left(\frac{R_{ij}+R_{ik}}{2}-R_{s}\right)}^{2}}f_{c}\left(R_{ij}\right)f_{c}\left(R_{ik}\right) \end{array}$$ (4)

The DP layer consists of a set of neural networks (which are called meta-NNs). Each meta-NN corresponds to a subset of the reference dataset and is trained to predict potential energy, atomic force, and RESP charges of the corresponding subset and two subsets that are closest to it.

**FIGURE 3:**
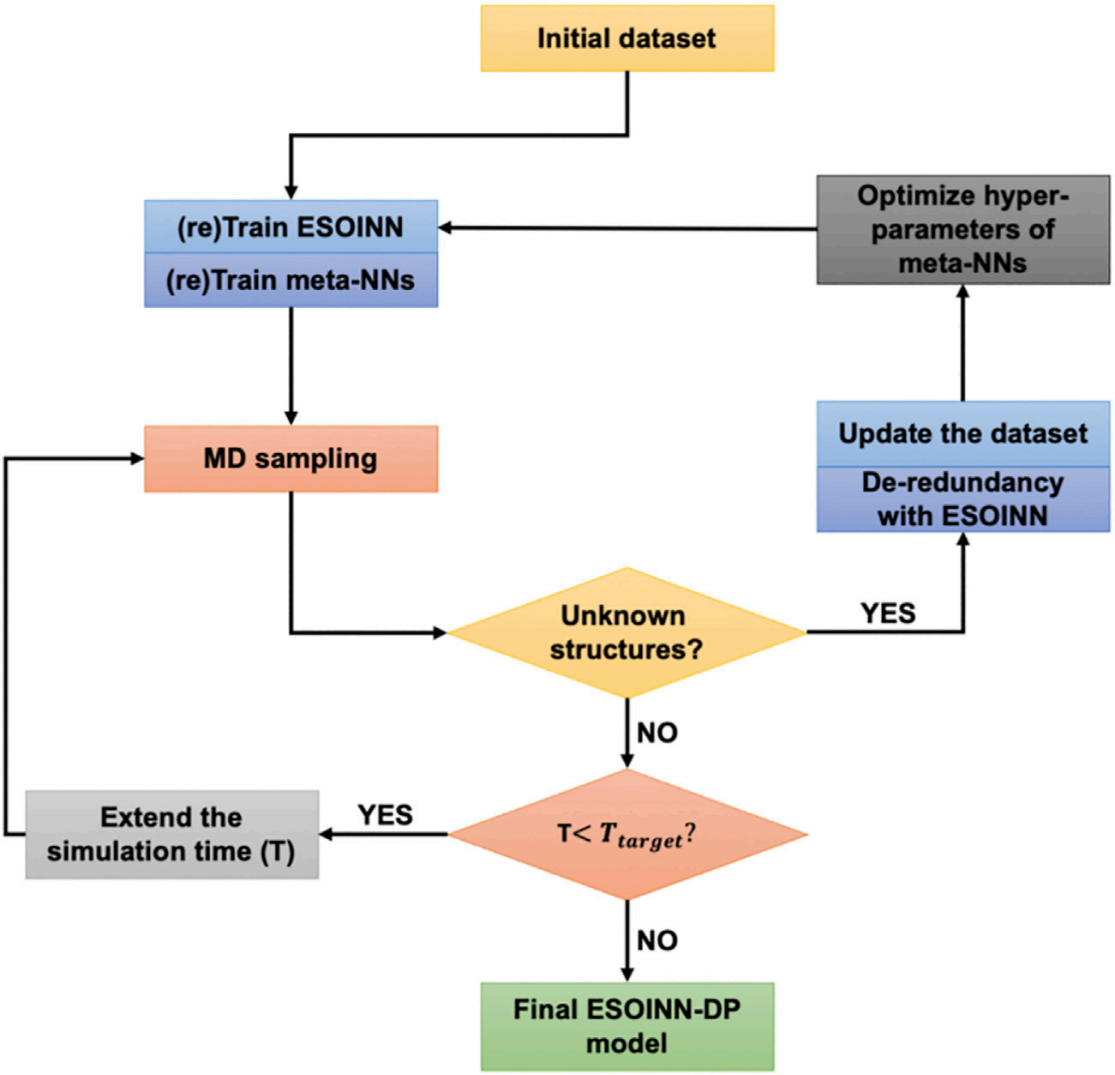
The training process of the NN potential.

By using ESOINN-DP, we developed NN potentials for four most common Zn^2+^ coordination modes (CCCC, CCCH, CCHH, HHHD) in zinc proteins. The training process of the NN potential is shown in Figure 3.

Take the CCCH coordination mode as an example. A typical protein which contains the CCCH-type MBG was firstly selected from the protein data bank (PDB ID: 2L30). Then a short (100 fs) MD simulation was performed, and MBGs were taken out from the trajectory every 5 fs. Potential energy, atomic forces, and RESP charges of these MBGs were calculated and taken as the initial dataset. After passing through the ESOINN layer, the initial training set is divided into several subsets, which are then learned by different meta-NNs. Then we use the current NN potential to re-run the MD simulation of the target system (starting from the initial structure) and extend the simulation time appropriately. As mentioned above, for each MBG in the training set, we have used three meta-NNs to learn its properties. When running MD, only one of them was used to drive the motion of atoms. But during the simulation, all of these three meta-NNs were used to predict the properties of MBG in each snapshot. In order to characterize the consistency of the three models, an error indicator ${\text{χ}}_{t}$ was defined:

$$\begin{array}{c} {\text{χ}}_{t}=max\left\|F_{net_{i},j}\left(R_{t}\right)-\langle F_{net_{i},j}\left(R_{t}\right)\rangle \right\| \end{array}$$ (5)

where $R_{t}$ denotes the given MBG and *j* is the index of atoms in MBG. On this basis, we can divide MBGs in the trajectory into three categories according to the value of ${\text{χ}}_{t}$:

$$\begin{array}{c} R_{t}=\{\begin{array}{cc} \text{known} & if0<{\text{χ}}_{t}\leq \delta \\ \text{questionable} & if\delta <{\text{χ}}_{t}\leq 2\delta \\ \text{unknown} & if\;{\text{χ}}_{t}>2\delta \end{array} \end{array}$$ (6)

Here δ is a pre-defined value, which represents the maximum error of NN potential we can tolerate. For the CCCH coordination mode, δ was set to 3.0 kcal/(mol⋅Å) The questionable MBGs will be sent to the ESOINN layer for de-redundancy, and their potential energy, atomic forces, and RESP charges will also be calculated and added to the reference dataset. It is worth mentioning that in the ESOINN-DP method, genetic algorithms will be used to re-adjust the hyper-parameters of meta-NN to save computing resources as much as possible while ensuring accuracy. Next, the ESOINN layer and meta-NNs will be retrained, and a new round of MD simulation will be performed. Using such an iterative process, we can gradually explore the target chemical space and keep the reference dataset as streamlined as possible. Finally, when the target length of MD is reached or no new questionable structure is detected, we get the final NN potential. Details of the ESOINN-DP method can be found in Ref 37.

### Computational Details

In this work, all QM calculations are performed with Gaussian 16at the M06-2X/SDD level. The M06-2X/SDD level was chosen because it has been proven be the most accurate one over other combinations of DFT functional and basis sets (Grauffel et al., 2018) in reproducing the structure of the zinc complex. The interaction between the MBG group and the rest of the protein is described by the electrostatic and van der Waals interaction with parameters obtained from the Amber ff14SB force field. To be consistent with Amber ff14SB, the electrostatic potential used to fit the RESP charge of MBG was obtained at the HF/6-31G* level. In the training of ESOINN, the maximum age of nodes was set to 10 and every 500 times inputs were defined as a learning cycle. The initial structural parameter of meta-NNs was set to [200,200,200]. We selected four different representative proteins for four coordination modes whose PDB ID are 1ZIN (CCCC), 2L30 (CCCH), 1AAY (CCHH), and 1HFS (HHHO). Before the MD simulations, we optimized the protein structure with the Amber ff14SB force field in a water ball with a radius of 25 Å. Then a 500ps heating simulation and a 5ns relaxation simulation were performed to fully relax a given protein structure. During the MD simulations with the Amber force field, structural constrains were added to the metal binding group to prevent the coordination geometry from being destroyed. After the pretreatment discussed above, the optimized system was used as the initial structure of MD simulations with NN potential.

## Result and Discussion

### Performance of the NN Potential

**FIGURE 4:**
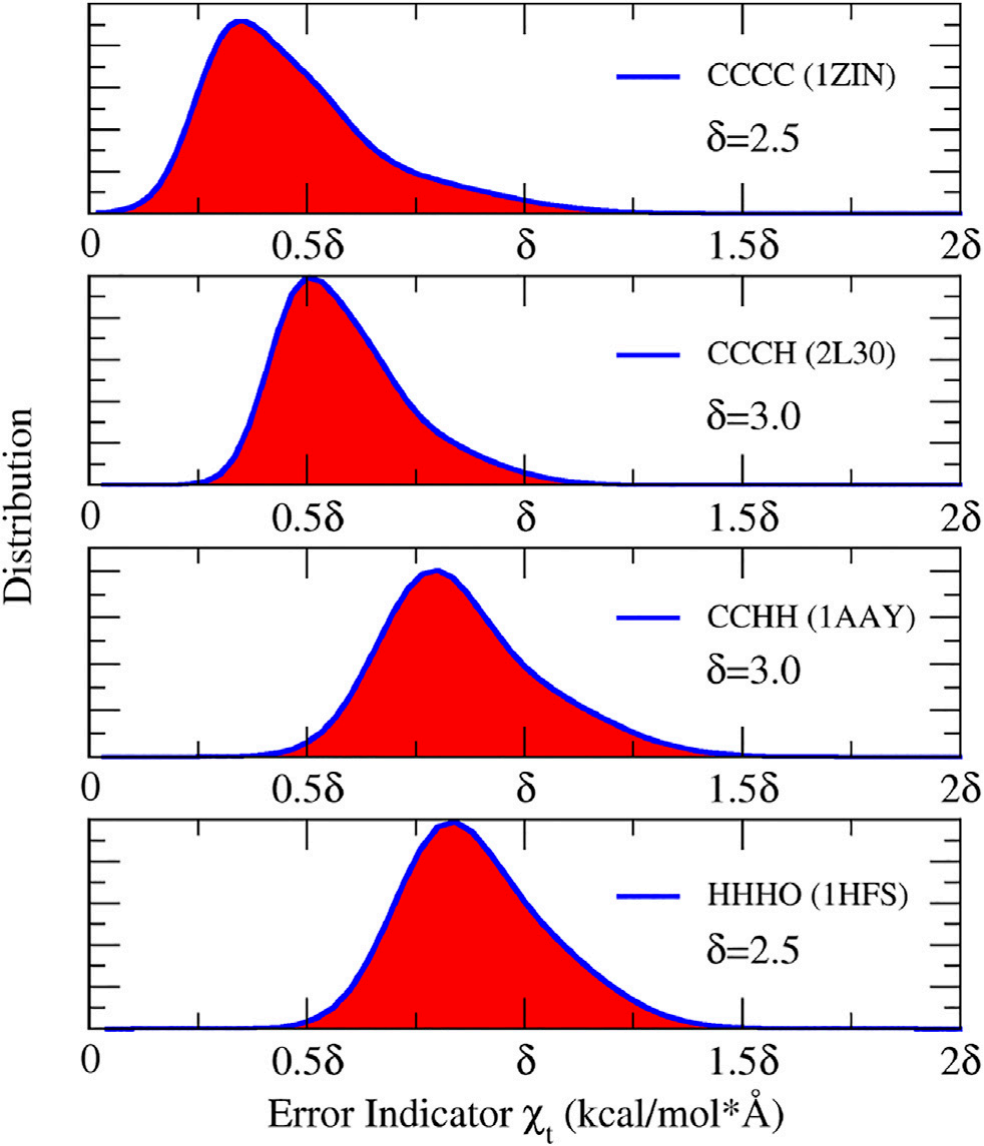
The distribution of error indicator $\chi_{t}$ in the four representative systems. The unit of δ is kcal/(mol⋅Å)

The performance of the NN potential on zinc-containing proteins with four coordination modes can be checked from Table 1. It can be seen that on both the training and test sets, the root mean square error (RMSE) of the potential energy for the CCHH type is the largest, which is only 1.78 kcal/mol. The RMSE of atomic forces for all systems are smaller than 1.8 kcal/(mol⋅Å). The good accuracy of NN potentials indicates that it can be readily used in the MD simulation. Then, a 1 ns MD simulation for each system was performed. During the simulation, the error indicator of the MBG was monitored. It should be pointed out that the error indicator represents the atomic force with the largest prediction error in each MBG. As shown in Figure 4, the error indicator of all the structures is within the range of (0, 2δ), which means that there are nearly no unknown structures in simulations and the trajectories are accurate. In fact, the maximum value of ${\text{χ}}_{t}$ in all of these four trajectories is only 4.68 kcal/(mol⋅Å). Therefore, we can confidently conclude that the reference dataset has covered the target chemical space, and the NN potential is reliable.

**TABLE 1:** The performance of NN potentials on four zinc-containing proteins with different coordination modes.

MBG type	PDB ID	Number of subsets in the ESOINN layer	Training set/test set			
			Size	RMSE of E (kcal/mol)	RMSE of F (kcal/(mol·Å))	RMSE of Q (e)
CCCC	1ZIN	7	11,900/1,200	1.43/1.29	1.53/1.43	0.04/0.05
CCCH	2L30	12	28,156/3,200	1.38/1.34	1.68/1.75	0.03/0.04
CCHH	1AAY	14	45,328/5,100	1.78/1.64	1.41/1.52	0.02/0.03
HHHO	1HFS	11	27,100/3,000	1.30/1.26	1.63/1.72	0.04/0.03

### Charge Distribution of Zinc Ion and Its Coordinated Atoms

In classical force fields such as Amber ff14SB, the charge of Zn is fixed at +2e. Thus, the electrostatic interactions between Zn^2+^ and the protein environment are very strong. However, it can be seen from the coordination field theory that after obtaining electrons shared by the ligand, the charge of the zinc ion cannot be so large. The positive divalent charge will cause the interaction between the zinc ions and other charged or polar groups in the protein to be seriously overestimated, resulting in unreliable MD simulation results. In NN/MM-RESP-MBG, the short-range polarization and charge transfer effects between Zn^2+^ and its ligands in MBG are fully considered by the neural network model. Meanwhile, we refitted the atomic charges of MBG, thereby avoiding the unphysical high charge of zinc ion, and making the interaction between MBG and MM regions more reasonable. Table 2 shows the average charge of zinc ion and its ligated atoms during the simulation.

**TABLE 2:** The averaged RESP charge of zinc ion and its ligated atoms in the MD simulation. The unit of charge is *e*.

1ZIN (CCCC)	Coordinated atoms	Zn^2+^	Sγ@C5	Sγ@C8	Sγ@C25	Sγ@C28
	NN/MM-RESP-MBG	1.25	−0.85	−0.87	−0.86	−0.84
	Amber	2	−0.88	−0.88	−0.88	−0.88
2L30 (CCCH)	Coordinated atoms	Zn^2+^	Sγ@C5	Sγ@C8	N^δ^@H37	Sγ@C40
	NN/MM-RESP-MBG	0.98	−0.87	−0.95	−0.41	−0.81
	Amber	2	−0.88	−0.88	−0.57	−0.88
1AAY (CCHH)	Coordinated atoms	Zn^2+^	Sγ@C5	Sγ@C10	N^ε^@H23	N^ε^@H27
	NN/MM-RESP-MBG	0.83	−0.82	−0.81	−0.37	−0.42
	Amber	2	−0.88	−0.88	−0.57	−0.57
1HFS (HHHO)	Coordinated atoms	Zn^2+^	N^ε^@H64	O^δ1^/O^δ2^@D66	N^ε^@H78	N^δ^@H92
	NN/MM-RESP-MBG	0.98	−0.51	−0.64/−0.87	−0.43	−0.38
	Amber	2	−0.57	−0.88	−0.57	−0.57

In the CCCC coordination mode, the average RESP charge of zinc ion (1.25 e) is the highest, which is still obviously lower than +2. The average RESP charge of the S atom is almost the same as the Amber charge. In the CCCH mode, the charge of zinc is 0.98 e, and it is clear that the charge of the coordinated N atom on histidine is obviously larger than the Amber charge. In the CCHH coordination mode, the average RESP charge of zinc ion is further reduced to 0.83 e. Finally, we analyze the protein contained in the HHHO coordination mode. In this system, one zinc ion forms coordination bonds with an aspartic residue and three histidine residues. Generally, when the carboxyl group coordinates with zinc ion, they can form either bidentate or single-dentate coordination modes, which depends on the interaction between the carboxyl group and the protein environment. In the 1HFS system, the coordination mode between the carboxyl group (E64) and zinc ion is single-dentate as the $O^{\delta 1}$ atom of E66 forms a hydrogen bond with Y68. The refitted RESP charge reflects this difference. The charge of the $O^{\delta 2}$ atom coordinated with zinc is significantly weakened due to the charge transfer, but the charge of the $O^{\delta 1}$ atom is still relatively large, which is necessary to maintain the hydrogen bond interaction. Furthermore, the charge of the zinc ion is reduced to 1.12 e. If we use the Amber charge to simulate this system, the carboxyl group will trend to form a bidentate coordination mode with the zinc ion, which will distort the protein structure. It should be pointed out that although the RESP charge is more suitable for calculating electrostatic interactions, its physical meaning cannot be guaranteed. The discussion of charge values here should be qualitative rather than quantitative. In future work, we will consider switching to a charge model that has more rigorous physical meaning and can accurately calculate the electrostatic interaction energy.

### Coordinate Geometry of MBGs

**FIGURE 5:**
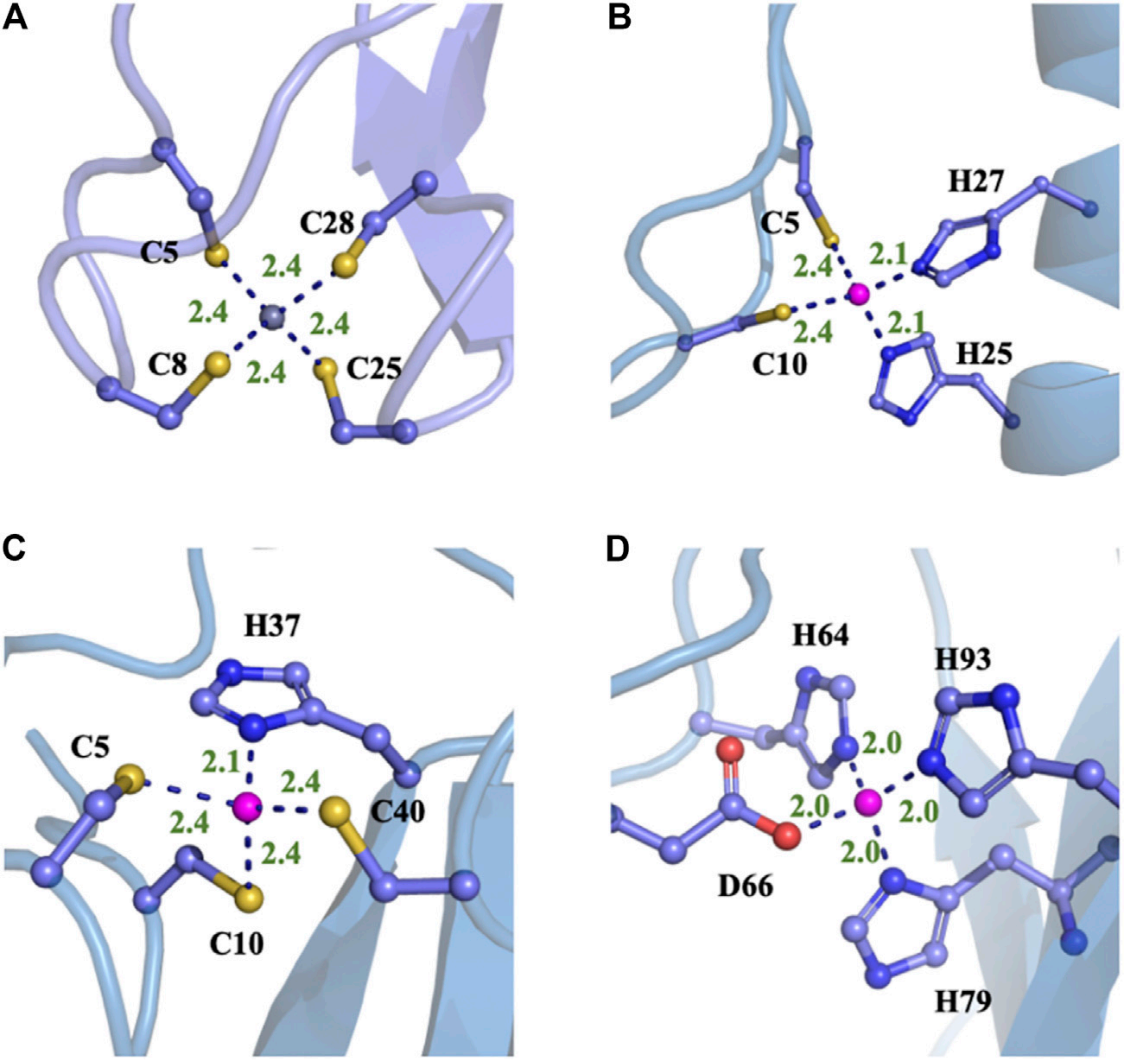
Typical structures of four different proteins with different MBGs after the MD simulations with NN/MM-RESP-MBG models. **(A)** CCCC type (PDB ID: 1ZIN), **(B)** CCHH type (PDB ID: 1AAY), **(C)** CCCH type (PDB ID: 2L30), and **(D)** HHHO (PDB ID: 1HFS).

Four typical MBG structures extracted from the MD simulation of four zinc proteins with the NN potential are shown in Figure 5. Detail coordination geometry parameters can found in Supplementary Figures S1–S8. Firstly, we analyzed the coordinate geometry of the MBG in protein 1ZIN. Table 3 shows the average value of coordinate bonds and angles in the MD trajectory along with the experimental values in the X-ray structure and value from the statistics of the same coordination modes in PDB. The distribution of bond length and angle can also be found in Supplementary Figures S1, S2. It can be seen that although the values obtained by MD simulation deviate slightly from the X-ray structure, they are all within the range of statistical values. Considering that the resolution of the experiment cannot reach the sub-Angstrom level, and the QM calculation itself still has room to be improved, the accuracy of the existing results has been very encouraging.

**TABLE 3:** Comparison of computed average distances and angles between zinc and its ligated atoms of different zinc proteins with experimental measurements, statistical values (Alberts et al., 1998), and results from MD simulations with the QPCT (Zhu et al., 2013) force field and QM/MM^13^ (all the bond lengths are in Angstroms and bond angles in degrees).

PDB ID	Zinc-ligand geometry	PDB survey	NN/MM-RESP-MBG	X-ray	QPCT Zhu et al. (2013)	QM/MM (50 ps) Zhu et al. (2013)
1ZIN (CCCC)	Zn-Sγ@C5	2.35 ± 0.09	2.41	2.33	2.37 ± 0.06	2.39 ± 0.08
	Zn-Sγ@C8	2.35 ± 0.09	2.39	2.3	2.36 ± 0.07	2.39 ± 0.09
	Zn-Sγ@C25	2.35 ± 0.09	2.4	2.32	2.37 ± 0.06	2.42 ± 0.09
	Zn-Sγ@C28	2.35 ± 0.09	2.41	2.33	2.36 ± 0.06	2.42 ± 0.08
	∠Sγ@C5-Zn-Sγ@C8	111 ± 8	107	114	N/A	N/A
	∠Sγ@C5-Zn-Sγ@C25	111 ± 8	116	106	114 ± 11	109 ± 6
	∠Sγ@C25-Zn-Sγ@C28	111 ± 8	111	112	N/A	N/A
1AAY (CCHH)	Zn-Sγ@C5	2.35 ± 0.09	2.35	2.29	2.29 ± 0.07	2.32 ± 0.06
	Zn-Sγ@C10	2.35 ± 0.09	2.34	2.29	2.30 ± 0.08	2.34 ± 0.07
	Zn-N^ε^@H23	2.05 ± 0.12	2.07	2.04	2.07 ± 0.12	2.12 ± 0.07
	Zn-N^ε^@H27	2.05 ± 0.12	2.09	2.04	2.08 ± 0.12	2.13 ± 0.07
	∠N^ε^@H23-Zn-N^ε^@H27	107 ± 8	97	105	101 ± 13	99 ± 7
	∠N^ε^@H23-Zn-Sγ@C5	109 ± 8	108	109	110 ± 13	108 ± 7
	∠Sγ@C5-Zn-Sγ@C10	111 ± 8	116	113	114 ± 10	114 ± 6
2L30 (CCCH)	Zn-Sγ@C5	2.35 ± 0.09	2.4	2.34	2.34 ± 0.07	2.34 ± 0.07
	Zn-Sγ@C8	2.35 ± 0.09	2.39	2.34	2.34 ± 0.08	2.34 ± 0.08
	Zn-N^δ^@H37	2.14 ± 0.09	2.15	2.01	2.17 ± 0.08	2.17 ± 0.08
	Zn-Sγ@C40	2.35 ± 0.09	2.35	2.34	2.34 ± 0.07	2.31 ± 0.08
	∠N^δ^@H37-Zn-Sγ@C40	109 ± 8	113	114	112 ± 7	108 ± 5
	∠Sγ@C5-Zn-Sγ@C8	111 ± 8	108	109	N/A	N/A
	∠Sγ@C8-Zn-Sγ@C40	111 ± 8	114	113	112 ± 7	114 ± 4
1HFS (HHHO)	Zn-N^ε^@H64	2.05 ± 0.12	2.02	1.83	1.94 ± 0.05	2.01 ± 0.08
	Zn-O^δ2^@D66	1.95 ± 0.08	1.97	2	1.97 ± 0.11	2.07 ± 0.09
	Zn-N^ε^@H79	2.05 ± 0.12	2.01	1.78	1.93 ± 0.09	1.98 ± 0.09
	Zn-N^δ^@H93	2.14 ± 0.09	2.06	2.01	1.95 ± 0.10	2.03 ± 0.09
	∠N^ε^@H64-Zn-O^δ2^@D66	107 ± 12	111	105	113 ± 6	107 ± 6
	∠N^ε^@H64-Zn-N^δ^@H79	112 ± 7	116	119	108 ± 7	111 ± 7
	∠N^ε^@H79-Zn-N^δ^@H93	112 ± 7	112	113	N/A	N/A

The calculated coordination geometry data and corresponding experimental values of the other three modes can be found in Table 3 and the supplementary materials. In most cases, the coordinate bond length and angle obtained from the MD trajectory are in good agreement with the experiment. However, for the N-Zn-N angle in the CCHH system, there is an obvious deviation. As can be seen from Supplementary Figure S4 and Table 3, the average value obtained from the trajectory is about 10° smaller than the value in the crystal structure and the statistical data. To further check the source of the error, we also compared current values with the QM/MM calculation of the same system in the previous work (Zhu et al., 2013), and unexpectedly found that they are very close to each other. The QM/MM calculation also employed the DFT method (B3LYP). However, it should be pointed out that when compared with the high-precision zinc complexes in the Cambridge structural database, the M06-2X/SDD level shows excellent accuracy (Grauffel et al., 2018). This has brought us certain difficulties in determining the source of the error. Although we will consider benchmarking higher-precision QM methods in future work, it cannot be ruled out that the experimental structure is problematic, after all, in the structural refinement process of both NMR and crystal diffraction experiments, an MD simulation with a traditional force field is employed. In addition, we also compared our results with that given by the QPCT force field. In most cases, the two are consistent. For the CCHH and HHHO geometries, the coordination bond length distribution obtained with NN/MM-RESP-MBG are closer to PDB bank statistic values than QPCT results as shown in Table 3.

**FIGURE 6:**
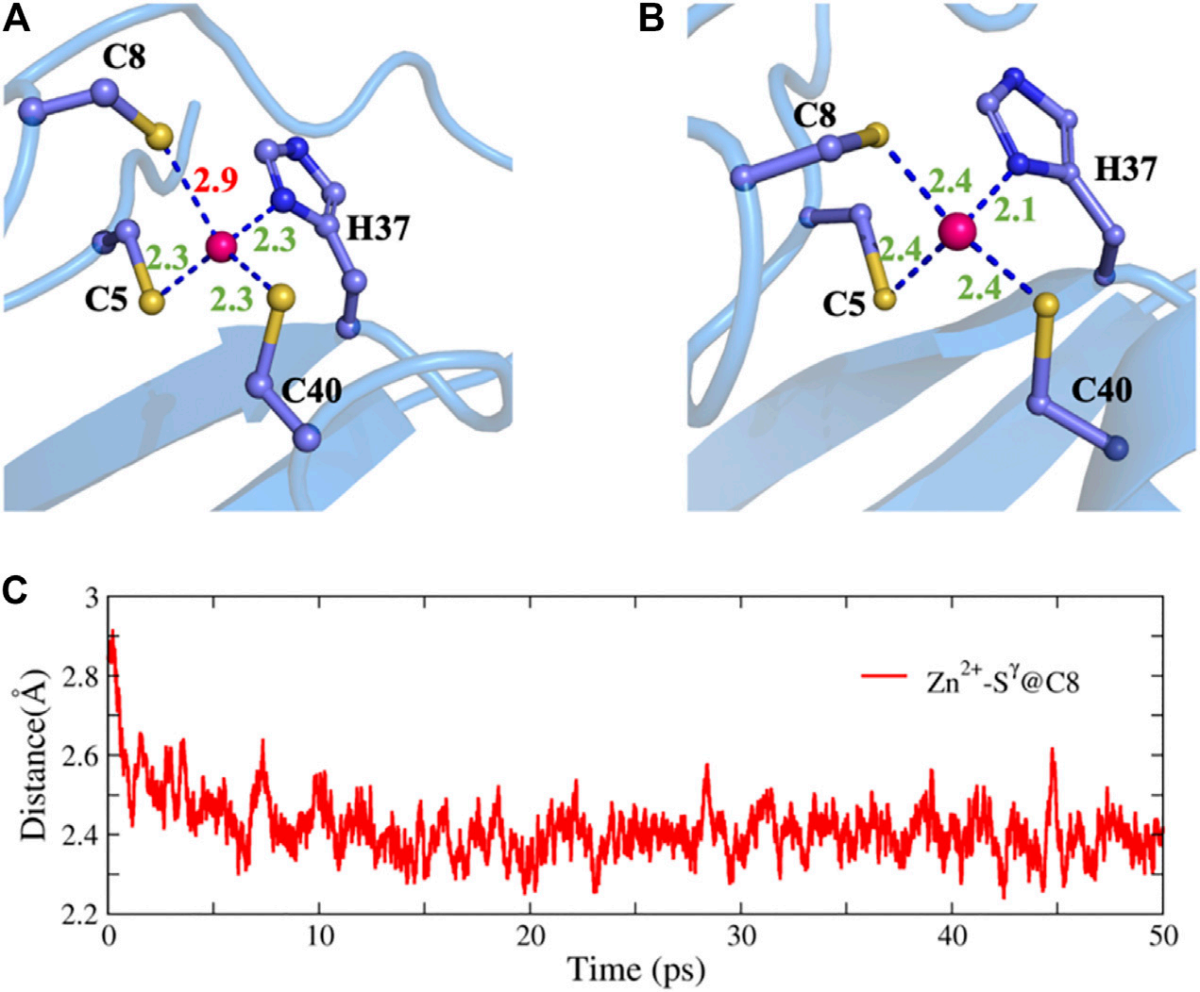
Structure refinement with NN potential for an ill structure of the 2L30 protein. **(A)** The ill structure, **(B)** the refined structure, and **(C)** the time evolution of the Zn-S distance.

To further test the ability of NN potentials, we also used it to refine two ill structures of zinc protein. The ill structure was produced by an MD simulation with an Amber ff14SB force field at 400 K. The first system is the 2L30 protein with an excessively long Zn-S bond. As shown in Figure 6, before optimization, the bond length of zinc ion and the $S^{\gamma }$ atom of C8 is almost 3.00 Å. Using this structure as the initial structure, we carried out a 50 ps MD simulation in combination with the NN potential. After the simulation, the length of this bond was restored to 2.40 Å and the regular tetrahedral coordination is well maintained. Another system is protein 1HFS with an excessively large N-Zn-O coordination angle and a distorted tetrahedral geometry. As can be seen from Supplementary Figure S9, MD with NN potential successfully repaired the structure within 5 ps.

**FIGURE 7:**
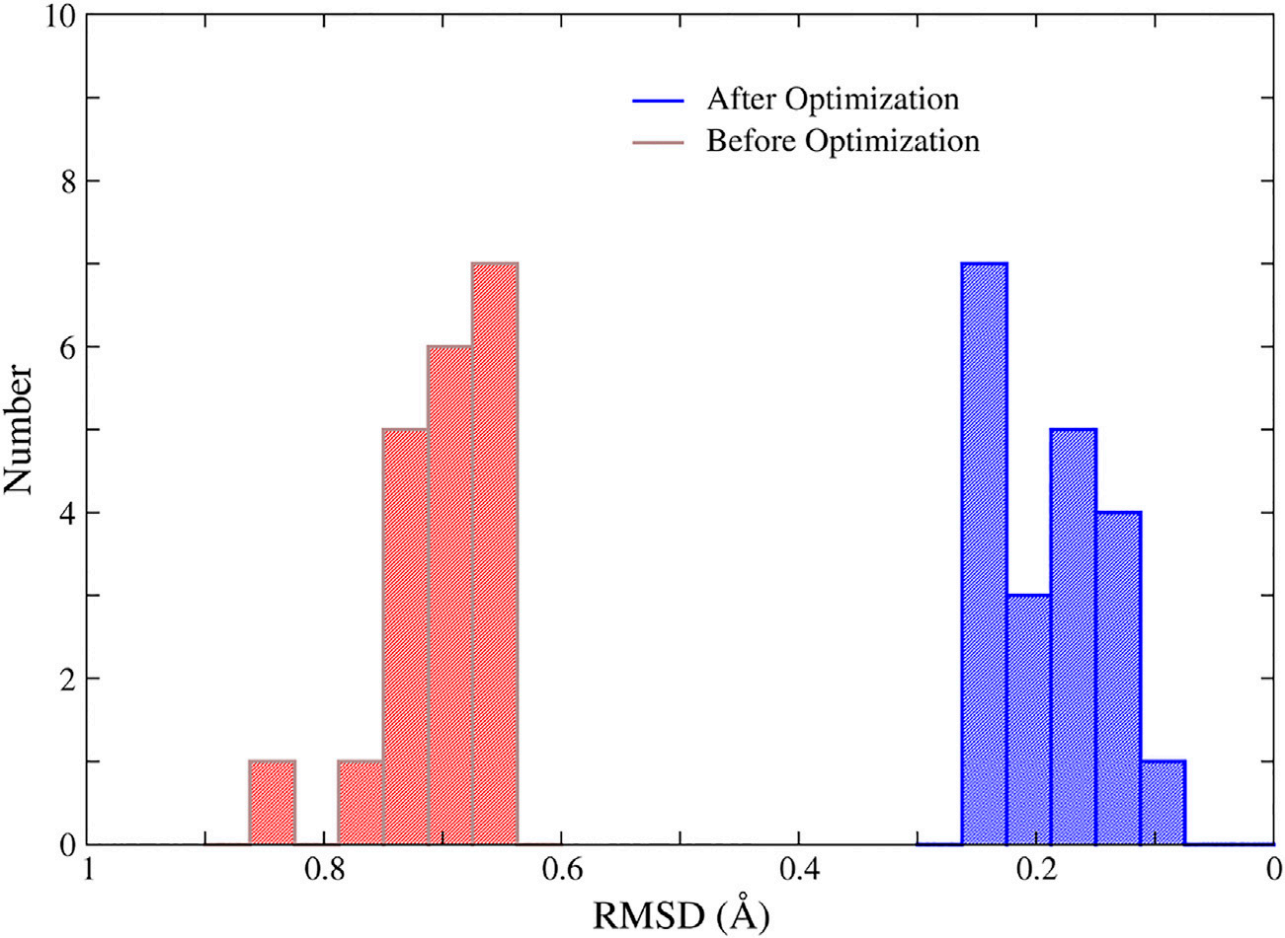
The RMSD of the MBGs in ill and optimized structures of the 1ZIN protein. The MBG of the X-ray structure was taken as the reference.

In addition, we randomly selected 20 structures of the 1ZIN protein with ill MBGs and optimized them with NN potentials and the conjugate gradient algorithm. The results are shown in Figure 7. Although the RMSD of the MBG structure before optimization does not look large, the actual structure is still very problematic due to the small number of atoms in the MBG. However, after simple optimization, the RMSD of all MBG structures is obviously reduced, with an average value of around 0.20 Å, which shows the great potential of NN potential for structural optimization.

To further demonstrate the transferability of the NN/MM-RESP-MBG method, 200 ps MD simulations were performed for other 14 zinc proteins with the CCHH and CCCC-type MBGs. Detailed MBG structures in the simulation are listed in Supplementary Tables S1, S2 and Supplementary Figures S10, S11. It can be seen clearly that all the results are in good agreement with the experimental values. This is predictable because the neural network models are trained for different MBGs and are not protein-specific.

## Conclusion and Outlook

In this work, NN potentials were automatically constructed by using the ESOINN-DP (https://github.com/tongzhugroup/ESOINN-DP) method for typical zinc proteins. For a given protein, the potential energy, atomic forces, and atomic charges of the metal-binding group are predicted by the neural network, while the interaction between MBG and the rest of the protein is treated by the classical force field. For the four most common zinc coordination modes in the protein, the NN predictions show great agreement with QM calculations. In addition, MD simulation and energy optimization with NN potential can be readily used for the structural refinement of MBG. Compared with classical molecular force fields, the neural network potential is not limited by the function form and complex parameterization process. All local quantum effects, especially the polarization and charge transfer can be accurately described. In addition, the computational efficiency of the NN potential is much faster than the QM and QM/MM calculations. For the zinc proteins studied in this work, it takes no more than 0.1 s for a single MD step on a common Linux server with a 16-core CPU and an NVIDIA GTX1080Ti GPU card. In fact, the efficiency still has great room to be improved as we did not optimize the code of MD simulation deeply.

Although the NN potential proposed in this work has the advantages of accuracy and efficiency over MM and QM methods, respectively, there are still some shortcomings. First, the polarization effect of the protein environment on MBG is not considered, and only the short-range polarization effect between the Zn^2+^ and the coordinated residue is included. Secondly, the neural network potential function model used in this work is trained with reference to the DFT calculation results. There is still room to improve the performance of DFT calculations. If the data can be labeled at a higher level, the accuracy of the NN potential will be further improved. Related research is being carried out in our laboratory. Despite these shortcomings, the current NN/MM-RESP-MBG models can be readily used to perform nanosecond-level MD simulations and structural optimization for zinc proteins. The algorithm proposed in this work can also be directly applied to proteins containing other metal ions.

## Data Availability

The raw data supporting the conclusion of this article will be made available by the authors, without undue reservation.
