# Supplementary material for: Automatically Constructed Neural Network Potentials for Molecular Dynamics Simulation of Zinc Proteins
Source: Front Chem. 2021 Jun 18;9:692200. doi: 10.3389/fchem.2021.692200 (PMC8249736; doi:10.3389/fchem.2021.692200)
Supplement: Supplementary file 1 [file DataSheet1.PDF]

Supplemental Information

Automatically Construct Neural Network Potentials for Molecular Dynamics Simulation of Zinc Proteins

Mingyuan Xu[1], Tong Zhu[1,2]* and John Z. H. Zhang[1,2,3,4]*

[1]*Shanghai Engineering Research Center of Molecular Therapeutics & New Drug Development, Shanghai Key Laboratory of Green Chemistry & Chemical Process, School of Chemistry and Molecular Engineering, East China Normal University, Shanghai 200062, China*

[2]*NYU-ECNU Center for Computational Chemistry at NYU Shanghai, Shanghai 200062, China*

[3]*Department of Chemistry, New York University, NY, NY 10003, USA*

[4]*Collaborative Innovation Center of Extreme Optics, Shanxi University, Taiyuan, Shanxi 030006, China*

*Correspondence should be addressed to: tzhu@lps.ecnu.edu.cn or john.zhang@nyu.edu

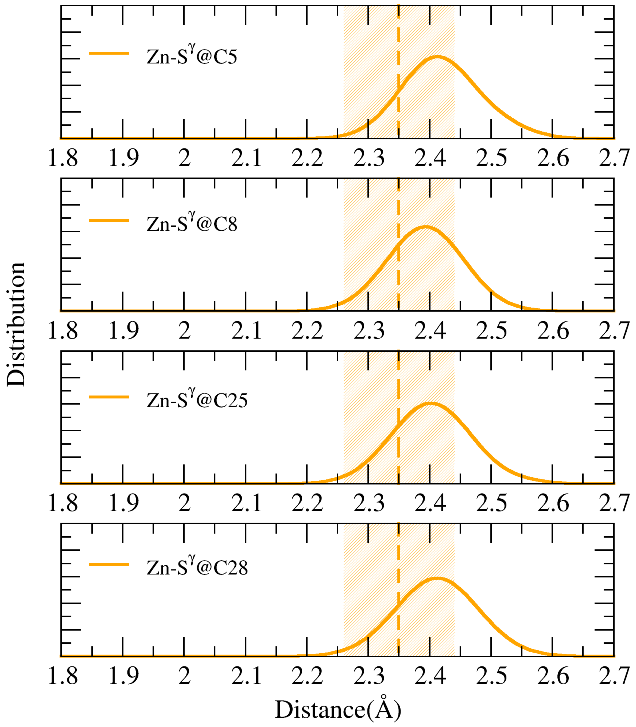

Figure S1. The distribution of coordination bond length in the MD simulation of the 2L30 protein. The dotted line and orange area represent the average value and distribution of statistic from the PDB database.

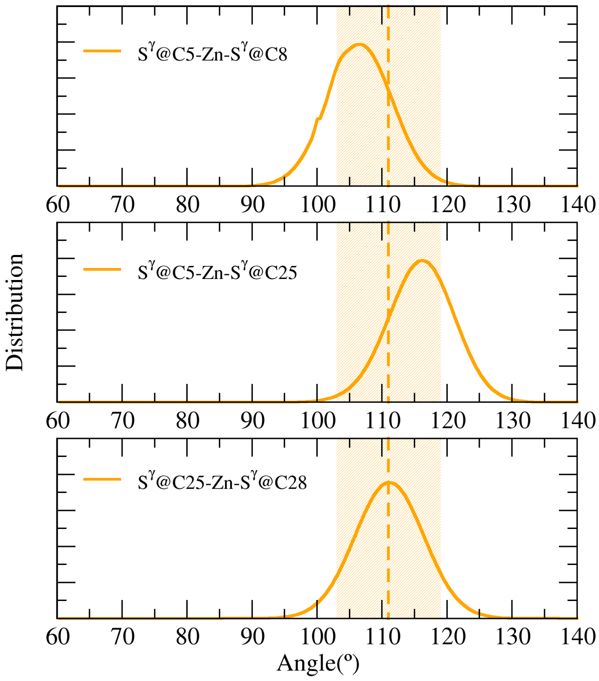

Figure S2. The distribution of coordination angle in the MD simulation of the 2L30 protein. The dotted line and orange area represent the average value and distribution of statistic from the PDB database.

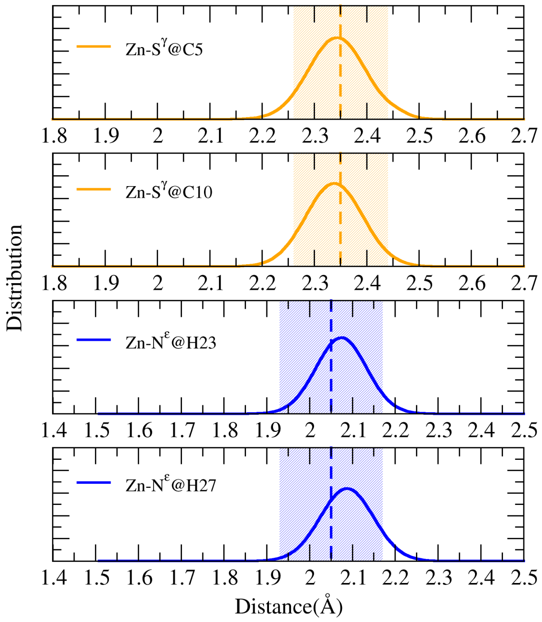

Figure S3. The distribution of coordination bond length in the MD simulation of the 1AAY protein. The dotted line and orange/blue area represent the average value and distribution of statistic from the PDB database.

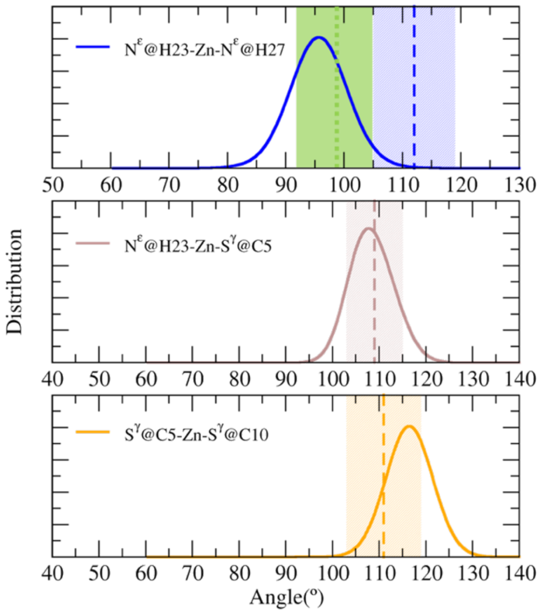

Figure S4. The distribution of coordination angle in the MD simulation of the 1AAY protein. The dotted line and orange/blue area represent the average value and distribution of statistic from the PDB database. The green dotted line and area represent the average value and distribution from the QM/MM simulation.

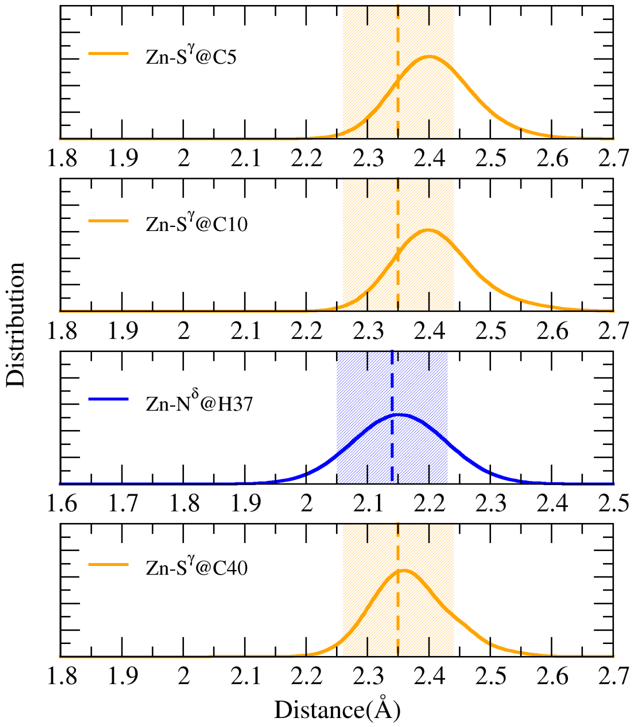

Figure S5. The distribution of coordination bond length in the MD simulation of the 2L30 protein. The dotted line and orange/blue area represent the average value and distribution of statistic from the PDB database.

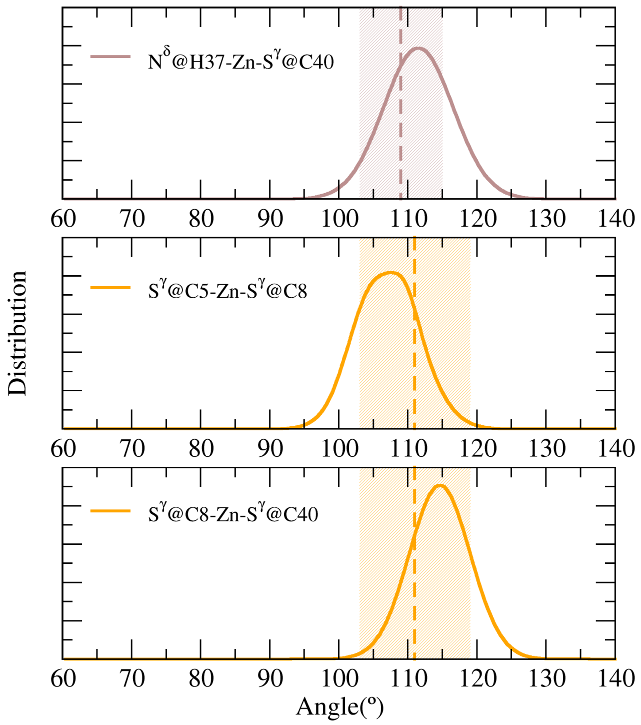

Figure S6. The distribution of coordination angle in the MD simulation of the 2L30 protein. The dotted line and orange/grey area represent the average value and distribution of statistic from the PDB database.

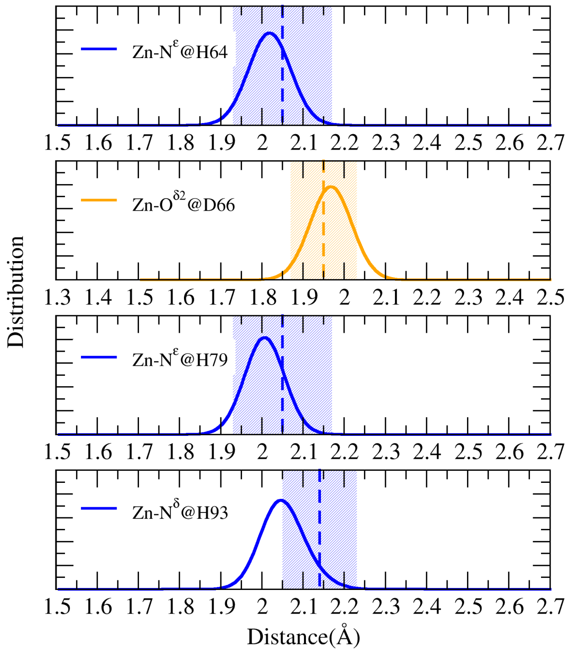

Figure S7. The distribution of coordination bond length in the MD simulation of the 1HFS protein. The dotted line and orange/blue area represent the average value and distribution of statistic from the PDB database.

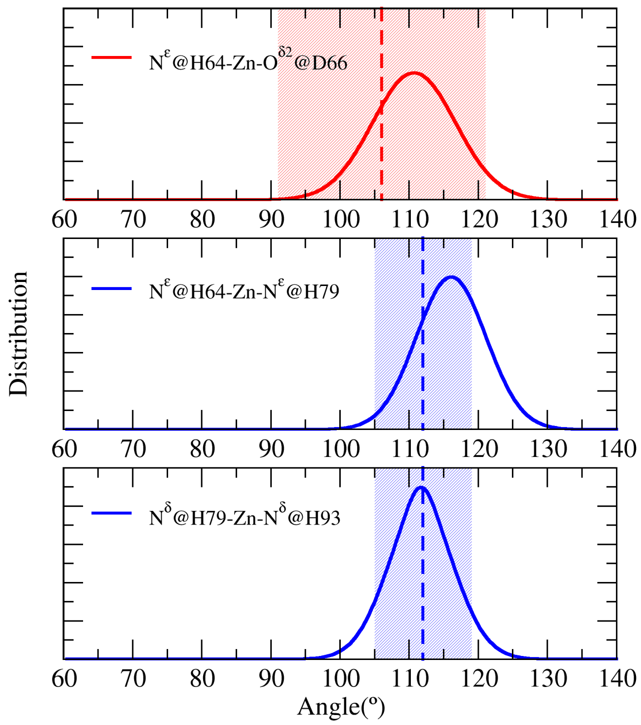

Figure S8. The distribution of coordination angle in the MD simulation of the 1HFS protein. The dotted line and red/blue area represent the average value and distribution of statistic from the PDB database.

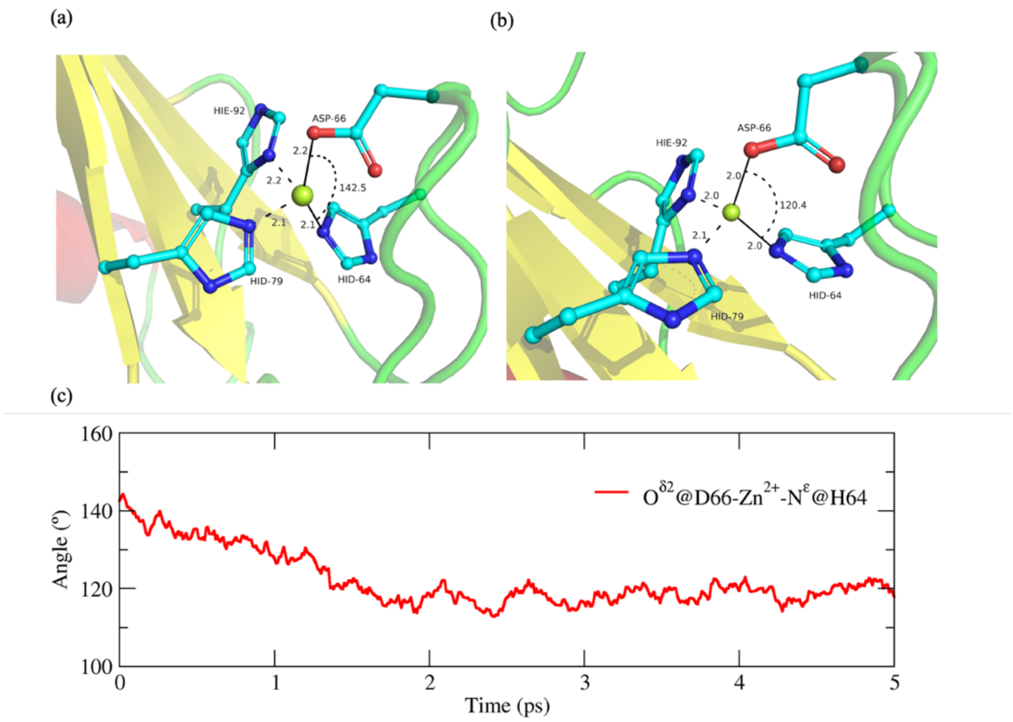

Figure S9. Structure refinement with NN potential for an ill structure of the 1HFS protein. (a) the ill structure, (b) the refined structure, (c) the time evolution of the O-ZN-N coordination angle.

Table S1 Comparison of computed average distances and angles between zinc and its ligated atoms in 6 different CCCC type metalloproteins with experimental and statistical values (all the bond lengths are in Angstroms and bond angles in degrees)

PDB ID	Zinc-ligand geometry	NN/MM-RESP-Metal	X-ray	PDB survey
	Zn-S^{γ}@C84	2.45±0.08	2.41	2.35 ± 0.09
	Zn-S^{γ}@C86	2.35±0.07	2.32	2.35 ± 0.09
	Zn-S^{γ}@C90	2.43±0.07	2.67	2.35 ± 0.09
1TWF	Zn-S^{γ}@C93	2.40±0.08	2.45	2.35 ± 0.09
	∠S^{γ}@C90-Zn-S^{γ}@C93	107±8	115	111 ± 8
	∠S^{γ}@C86-Zn-S^{γ}@C93	109±6	132	111 ± 8
	∠S^{γ}@C84-Zn-S^{γ}@C86	107±8	117	111 ± 8
	Zn-S^{γ}@C151	2.42±0.08	2.44	2.35 ± 0.09
	Zn-S^{γ}@C154	2.37±0.08	2.29	2.35 ± 0.09
	Zn-S^{γ}@C171	2.39±0.07	2.32	2.35 ± 0.09
1U5K	Zn-S^{γ}@C174	2.42±0.08	2.41	2.35 ± 0.09
	∠S^{γ}@C154-Zn-S^{γ}@C171	106±8	109	111 ± 8
	∠S^{γ}@C171-Zn-S^{γ}@C174	108±6	108	111 ± 8
	∠S^{γ}@C84-Zn-S^{γ}@C86	115±8	112	111 ± 8
	Zn-S^{γ}@C12	2.42±0.08	2.33	2.35 ± 0.09
	Zn-S^{γ}@C15	2.37±0.08	2.32	2.35 ± 0.09
	Zn-S^{γ}@C26	2.45±0.07	2.33	2.35 ± 0.09
2BX9	Zn-S^{γ}@C29	2.45±0.08	2.33	2.35 ± 0.09
	∠S^{γ}@C26-Zn-S^{γ}@C29	106±8	109	111 ± 8
	∠S^{γ}@C12-Zn-S^{γ}@C29	113±6	106	111 ± 8
	∠S^{γ}@C15-Zn-S^{γ}@C29	115±5	93	111 ± 8
	Zn-S^{γ}@C3	2.23±0.08	2.44	2.35 ± 0.09
	Zn-S^{γ}@C6	2.32±0.08	2.29	2.35 ± 0.09
	Zn-S^{γ}@C24	2.33±0.07	2.32	2.35 ± 0.09
2UVT	Zn-S^{γ}@C27	2.35±0.08	2.41	2.35 ± 0.09
	∠S^{γ}@C6-Zn-S^{γ}@C24	113±8	117	111 ± 8
	∠S^{γ}@C6-Zn-S^{γ}@C27	108±6	110	111 ± 8
	∠S^{γ}@C3-Zn-S^{γ}@C6	110±8	110	111 ± 8
	Zn-S^{γ}@C7	2.39±0.08	2.36	2.35 ± 0.09
	Zn-S^{γ}@C25	2.43±0.08	2.29	2.35 ± 0.09
	Zn-S^{γ}@C27	2.38±0.07	2.18	2.35 ± 0.09
3FQM	Zn-S^{γ}@C48	2.35±0.08	2.43	2.35 ± 0.09
	∠S^{γ}@C7-Zn-S^{γ}@C27	115±8	108	111 ± 8
	∠S^{γ}@C48-Zn-S^{γ}@C27	117±6	110	111 ± 8
	∠S^{γ}@C7-Zn-S^{γ}@C48	105±9	110	111 ± 8
3GLS	Zn-S^{γ}@C135	2.42±0.08	2.43	2.35 ± 0.09
	Zn-S^{γ}@C138	2.38±0.08	2.43	2.35 ± 0.09

	Zn-S^{γ}@C159	2.40±0.07	2.39	2.35 ± 0.09
	Zn-S^{γ}@C162	2.38±0.08	2.23	2.35 ± 0.09
	∠S^{γ}@C138-Zn-S^{γ}@C159	108±8	115	111 ± 8
	∠S^{γ}@C159-Zn-S^{γ}@C162	108±6	106	111 ± 8
	∠S^{γ}@C135-Zn-S^{γ}@C138	110±8	102	111 ± 8

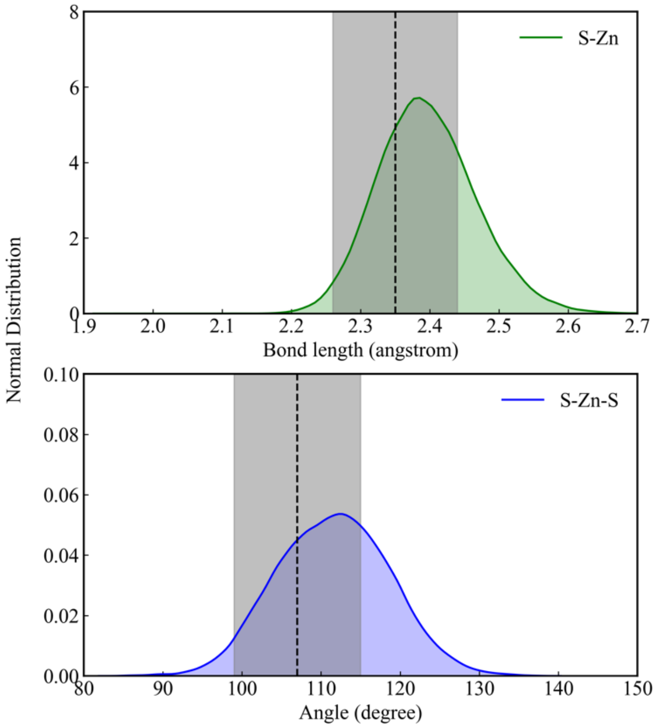

Figure S10. The distribution of coordination bond length in the MD simulation of 6 different CCCC type metalloprotein (PDB ID: 1TWF,1U5K,2BX9,2UVT,3FQM and 3GLS). The dotted line and grey area represent the average value and distribution of statistic from the PDB database.

Table S2 Comparison of computed average distances and angles between zinc and its ligated atoms in 8 different CCHH type metalloproteins with experimental and statistical values (all the bond lengths are in Angstroms and bond angles in degrees)

PDB ID	Zinc-ligand geometry	NN/MM-RESP-Metal	X-ray	PDB survey
2ELN	Zn-S^{γ}@C12	2.34±0.08	2.36	2.35 ± 0.09
	Zn-S^{γ}@C17	2.33±0.08	2.32	2.35 ± 0.09
	Zn-N^{ε}@H30	2.10±0.07	1.98	2.05 ± 0.12
	Zn-N^{ε}@H34	2.11±0.09	1.92	2.05 ± 0.12
	∠N^{ε}@H30-Zn-N^{ε}@H34	105±5	110	107 ± 8
	∠N^{ε}@H34-Zn-S^{γ}@C12	109±6	104	109 ± 8
	∠S^{γ}@C5-Zn-S^{γ}@C10	125±5	114	111 ± 8
2ELO	Zn-S^{γ}@C12	2.33±0.07	2.24	2.35 ± 0.09
	Zn-S^{γ}@C15	2.31±0.06	2.21	2.35 ± 0.09
	Zn-N^{ε}@H28	2.14±0.07	2.08	2.05 ± 0.12
	Zn-N^{ε}@H33	2.06±0.09	2.02	2.05 ± 0.12
	∠N^{ε}@H28-Zn-N^{ε}@H33	105±5	110	107 ± 8
	∠N^{ε}@H33-Zn-S^{γ}@C12	95±6	101	109 ± 8
	∠S^{γ}@C12-Zn-S^{γ}@C15	122±6	116	111 ± 8
2ENA	Zn-S^{γ}@C15	2.37±0.08	2.33	2.35 ± 0.09
	Zn-S^{γ}@C18	2.35±0.06	2.19	2.35 ± 0.09
	Zn-N^{ε}@H31	2.09±0.07	2.09	2.05 ± 0.12
	Zn-N^{ε}@H35	2.08±0.06	1.9	2.05 ± 0.12
	∠N^{ε}@H31-Zn-N^{ε}@H35	105±5	110	107 ± 8
	∠N^{ε}@H33-Zn-S^{γ}@C15	95±6	101	109 ± 8
	∠S^{γ}@C15-Zn-S^{γ}@C18	123±6	116	111 ± 8
2EOQ	Zn-S^{γ}@C15	2.37±0.08	2.33	2.35 ± 0.09
	Zn-S^{γ}@C18	2.35±0.06	2.19	2.35 ± 0.09
	Zn-N^{ε}@H31	2.11±0.07	2.09	2.05 ± 0.12
	Zn-N^{ε}@H35	2.08±0.06	1.9	2.05 ± 0.12
	∠N^{ε}@H31-Zn-N^{ε}@H35	97±6	94	107 ± 8
	∠N^{ε}@H31-Zn-S^{γ}@C18	110±7	101	109 ± 8
	∠S^{γ}@C15-Zn-S^{γ}@C18	113±6	118	111 ± 8
2EOR	Zn-S^{γ}@C15	2.32±0.07	2.39	2.35 ± 0.09
	Zn-S^{γ}@C18	2.36±0.06	2.19	2.35 ± 0.09
	Zn-N^{ε}@H31	2.11±0.07	2.1	2.05 ± 0.12
	Zn-N^{ε}@H35	2.06±0.06	2.1	2.05 ± 0.12
	∠N^{ε}@H31-Zn-N^{ε}@H35	95±6	112	107 ± 8

	∠N^{ε}@H35-Zn-S^{γ}@C18	114±7	119	109 ± 8
	∠S^{γ}@C15-Zn-S^{γ}@C18	108±6	108	111 ± 8
2EP0	Zn-S^{γ}@C15	2.32±0.07	2.39	2.35 ± 0.09
	Zn-S^{γ}@C18	2.34±0.06	2.39	2.35 ± 0.09
	Zn-N^{ε}@H31	2.12±0.07	1.97	2.05 ± 0.12
	Zn-N^{ε}@H35	2.09±0.06	1.9	2.05 ± 0.12
	∠N^{ε}@H31-Zn-N^{ε}@H35	103±6	117	107 ± 8
	∠N^{ε}@H35-Zn-S^{γ}@C15	107±7	107	109 ± 8
	∠S^{γ}@C15-Zn-S^{γ}@C18	116±6	110	111 ± 8
2EPS	Zn-S^{γ}@C15	2.34±0.06	2.39	2.35 ± 0.09
	Zn-S^{γ}@C18	2.33±0.07	2.39	2.35 ± 0.09
	Zn-N^{ε}@H31	2.12±0.07	2.01	2.05 ± 0.12
	Zn-N^{ε}@H36	2.07±0.06	1.92	2.05 ± 0.12
	∠N^{ε}@H31-Zn-N^{ε}@H36	99±6	107	107 ± 8
	∠N^{ε}@H31-Zn-S^{γ}@C18	105±7	107	109 ± 8
	∠S^{γ}@C15-Zn-S^{γ}@C18	122±6	118	111 ± 8
2YTF	Zn-S^{γ}@C15	2.37±0.06	2.29	2.35 ± 0.09
	Zn-S^{γ}@C18	2.33±0.07	2.27	2.35 ± 0.09
	Zn-N^{ε}@H31	2.10±0.07	2.1	2.05 ± 0.12
	Zn-N^{ε}@H35	2.11±0.06	1.92	2.05 ± 0.12
	∠N^{ε}@H31-Zn-N^{ε}@H35	102±6	108	107 ± 8
	∠N^{ε}@H35-Zn-S^{γ}@C15	107±7	113	109 ± 8
	∠S^{γ}@C15-Zn-S^{γ}@C18	111±6	111	111 ± 8

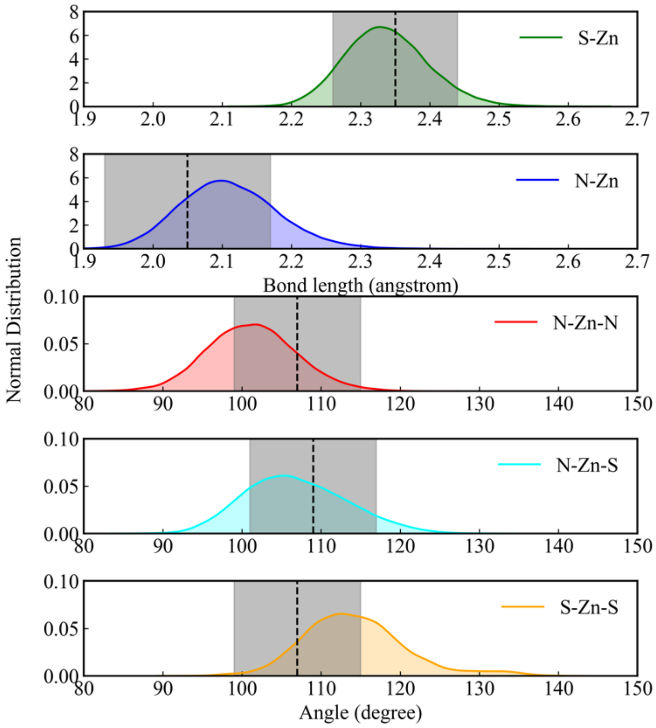

Figure S11. The distribution of coordination bond length in the MD simulation of 8 different CCHH type metalloprotein (PDB ID:2ELN,2ELO,2ENA,2EOQ,2EOR,2EP0,2EPS,2YTF). The dotted line and grey area represent the average value and distribution of statistic from the PDB database.
